# Spatial and temporal patterns of Lyme Neuroborreliosis on Funen, Denmark from 1995–2014

**DOI:** 10.1038/s41598-020-64638-5

**Published:** 2020-05-08

**Authors:** Amalie Muus Andreasen, Petter Bart Dehlendorff, Fredrikke Christie Knudtzen, Rene Bødker, Lene Jung Kjær, Sigurdur Skarphedinsson

**Affiliations:** 1 Clinical Institute, University of Southern Denmark, Campusvej 55, 5230 Odense M, Denmark; 2 Department of Infectious Diseases, CCEVI, Clinical Centre of Emerging and Vector-borne Infections, Odense University Hospital, Winsloews vej 4, 5000 Odense C, Denmark; 3 Department of Veterinary and Animals Sciences, University of Copenhagen, Frederiksberg, Denmark

**Keywords:** Central nervous system infections, Epidemiology

## Abstract

In Europe, Lyme neuroborreliosis (LNB) is the most severe manifestation of Lyme borreliosis and has recently been added to the communicable disease surveillance list for EU/EEA by the European Commission. In Northern Europe, LNB is primarily caused by the spirochete *Borrelia garinii* and transmitted by the tick *Ixodes ricinus*. This Danish observational epidemiologic case-control study includes every identified LNB patient (n = 401) on Funen, Denmark, from 1995-2014. We display spatial and temporal LNB incidence variation, seasonal distribution of cases and local spatial case clustering. Seasonal patterns show LNB symptom-onset peaking in July and a significant seasonal difference in number of cases (p < 0.01). We found no significant change in seasonality patterns over time when dividing the study period into 5-year intervals. We identified a significant local geographical hot-spot of cases with a relative risk of 2.44 (p = 0.013). Analysis revealed a significantly shorter distance to nearest forest for cases compared with controls (p < 0.001). We present a novel map of the focal geographical distribution of LNB cases in a high endemic borreliosis area. Continued studies of case clustering in the epidemiology of LNB are of key importance in guiding intervention strategies.

## Introduction

Lyme Borreliosis (LB) is the most common tick-borne infection in Denmark and in Europe^1–3^. It is a spirochetal infection caused by *Borrelia burgdorferi* sensu lato (sl), which in Denmark is transmitted by the tick *Ixodes ricinus*^2^. This tick species is found throughout the country, but it is most abundant in the eastern and central parts of Denmark^1^. LB can manifest with neurological symptoms, called Lyme neuroborreliosis (LNB), the most severe form of LB. A previous study has strongly suggested a correlation between variations in tick density and LNB incidence in Denmark^1^.

Primarily, four genospecies of *B. burgdorferi* sl are known to be associated with human disease. *B. burgdorferi* sensu stricto (ss), *B. afzelii*, *B. garinii* and *B. bavariensis*. In Europe, the predominant species are *B. afzelii*, *B. garinii* and *B. bavariensis*^3,4^.

Infections with different genospecies often result in different clinical manifestations. *B. garinii* primarily causes LNB with symptoms like lymphocytic meningitis, painful radiculitis and cranial neuropathy (particularly facial nerve palsy). *B. afzelii* is mostly associated with skin manifestations such as erythema migrans and acrodermatitis chronica atrophicans^3,4^. Different *Borrelia* genospecies have different preferred reservoir hosts, and thus the distribution of clincal manifestations may vary. The incidence of LB in Europe has increased over the past few years^2^. In a recognition of this, the European Commision has in 2018 amended LNB to the communicable disease surveillance list^5^, in an effort to monitor the epidemiology in order to support measures to prevent and control the disease and the following complications. In Denmark, the LNB incidence was found to be 3.2/100,000 population when the national microbiology database (MiBa) was used for surveillance^6^, while our research group have found a higher incidence of 4.76/100.000 in the area of Funen^7^.

Humans living in regions with competent hosts of *I. ricinus* are at higher risk of disease, as these may serve as reservoirs hosts for various pathogens that can be transmitted by tick bites to humans^8^. Although the distribution and abundance of ticks are highly impacted by climate and landscape^9,10^, abundance of host species also affect the presence and abundance of ticks^10^. Among other species, the European roe deer (*Capreolus capreolus*) is an important tick host and previous studies have found a correlation between tick abundance and roe deer abundance at a local scale. Thus, changes in the roe deer population may alter the number of ticks in the following seasons^11^. Local variations in reservoir host animal numbers can however also affect local difference in *Borrelia* genospecies domination^3,12^. The risk of acquiring LNB is thus a complex interplay between *Borrelia* reservoir host distribution and tick abundance.

The primary objectives of this observational study were to (1) describe both the spatial and temporal LNB incidence variation, and examine any change in seasonal distribution over the last 20 years, and (2) identify potential spatial patterns of LNB-cases on Funen, and quantify difference in distance to nearest forest between cases and controls based on home addresses.

## Method

### Study population

**Figure 1:**
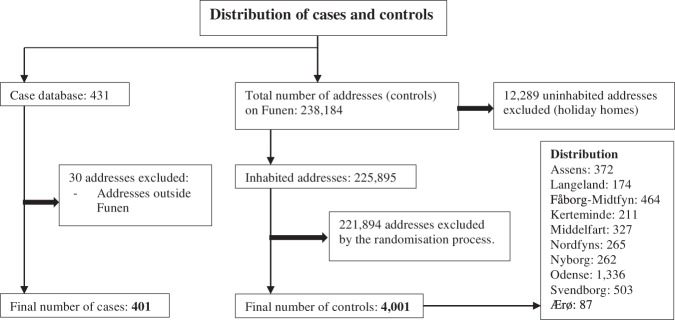
Flowchart of Lyme Neuroborreliosis case (n = 401) and control (n = 4001) address distribution on Funen, Denmark 1995–2014, included in the study. Bold arrows indicate exclusion.

A former study of every available patient chart from Funen, Denmark in the period 01.01.1995 to 31.12.2014, uncovered 431 patients with a LNB diagnosis^7^. A diagnosis was made if the patient had clinical symptoms of LNB and a positive *Borrelia* intrathecal antibody index test (IgM and/or IgG) performed at the Department of Microbiology, Odense University Hospital^6^. Of these 431 patients, 401 were included in this study (Fig. 1).

We extracted case addresses and the date of symptom-onset from the case database. At extraction, case addresses were scrambled, by randomly changing the house number to either +1, no change, or −1, due to guidelines regarding clinical research issued by the Danish National Committee on Health Research Ethics^13^.

The control addresses were obtained by extracting a list of every residential address in each of the 10 municipalities of Funen from the publicly available national address database^14^. Among the 238,184 extracted control addresses, we randomly chose 4001 using the RAND-function in Excel (Fig. 1).

### Statistical analysis

#### Incidence and regional mapping

The annual LNB incidence rate (IR) of the region of Funen was calculated from the publicly available municipality population numbers^15^. However, as the official population numbers from 1995-96 were not available, IR could only be calculated from 1997-2014. The Edwards test was used to test for seasonality in month of symptom debut^16^. To test for significant differences in monthly distribution of cases in four 5-year time periods (1995–1999, 2000–2004, 2005–2009 and 2010–2014), the Kruskal-Wallis test for non-normally distributed data was used. The analyses were carried out using STATA version 15.0. A p-value <0.05 was considered statistically significant.

#### Local spatial clustering

We used ArcMap 10.1 ESRI. Redlands, CA, a program used to manage geographic data^17^, and to run an initial IDW interpolation to help us to visualize areas with potential high or low clustering of both cases and controls. The search radius was set to 5000 meters, as Funen is of limited size with many small forest areas, and we deemed this a reasonable distance that an individual would regularly travel away from their home address (going for a walk, walking the dog).

We performed a purely spatial analysis to test for and to identify local level clustering using the software SaTScan^18^ after transforming the address coordinates to the Universal Transverse Mercator coordinate system (UTM). The analysis included scanning for both circular and elliptic shaped clusters, containing significantly high/low rates (hot/cold spots) of cases, using the Bernoulli probability model^19^.

#### Distance to the nearest forest

We created a new 1 ×1 km raster layer of the CORINE Land Cover classification^20^ with only forested areas on Funen (Supplement S1). For each case and control address, we used the Spatial Analyst tool in ArcMap ESRI. Redlands, CA to calculate the Euclidian distance to the nearest forest pixel for both cases and controls. To account for spatial autocorrelation of data points, we created a 3×4 grid and overlayed it to our study area. We then extracted grid id for each of the cases and controls, and used this grid id as a random effect in a mixed model logistic regression^21^(see Supplement S2) to identify any increase in the probability of becoming a case rather than a control when moving one km closer to a forest area.

### Ethical considerations

This study was approved by the Danish Data Protection Agency (j.nr. 2008-58-0035) and the Danish Health and Medicines Authority (j.nr. 3-3013-631/1/).

## Results

### Seasonal variation of symptom-onset

**Figure 2:**
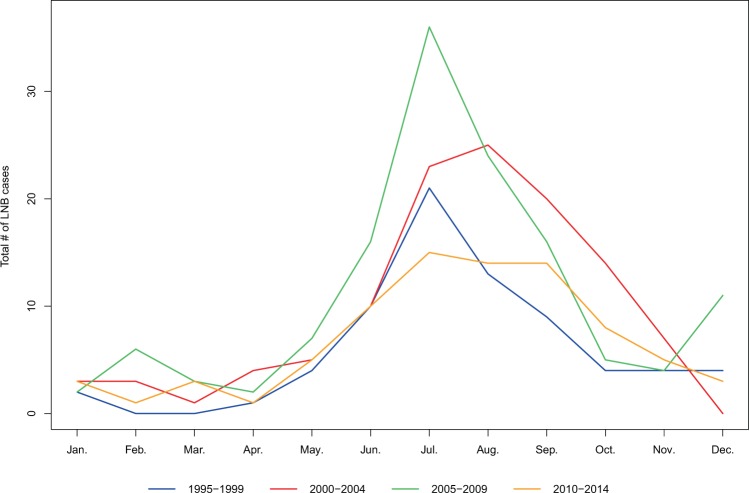
Seasonal variation of LNB cases in 5-year intervals (1995–99, 2000–04, 2005–09 and 2010–14).

We found a seasonal pattern, with the number of cases starting to increase in May and peaking in July. There was a statistically significant seasonal variability of cases (p < 0.01). Divided into four seasons the majority had symptom debut in late summer in July, August and September (n = 230, 57%) compared to only 6.7% (n = 27) in the months of January, February and March (p < 0.001). When displaying seasonal variation in 5-year intervals we found the same pattern with symptom-onset peaking in July based on total numbers of cases, except in 2000–2004 where symptom-onset peaked in August, by a margin of two cases (Fig. 2). We found no statistically significant differences in monthly distribution of cases between the four 5-year intervals (all p-values > 0.05, results displayed in Supplement S3).

### Incidence rate and regional mapping

**Figure 3:**
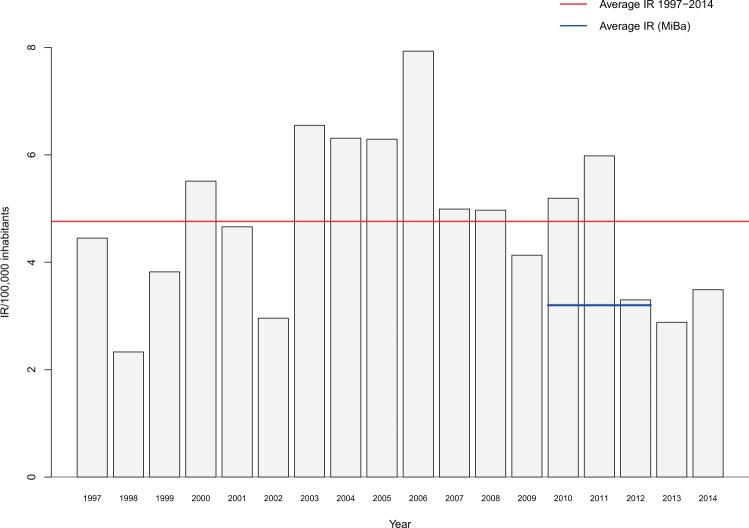
Annual incidence rate (IR) and average incidence of Lyme Neuroborreliosis cases (n = 401) on Funen, Denmark, 1997–2014. MiBa average IR, Denmark 2010–2012. National microbiology database (MiBa), Dessau *et al*. 2012.

**Figure 4:**
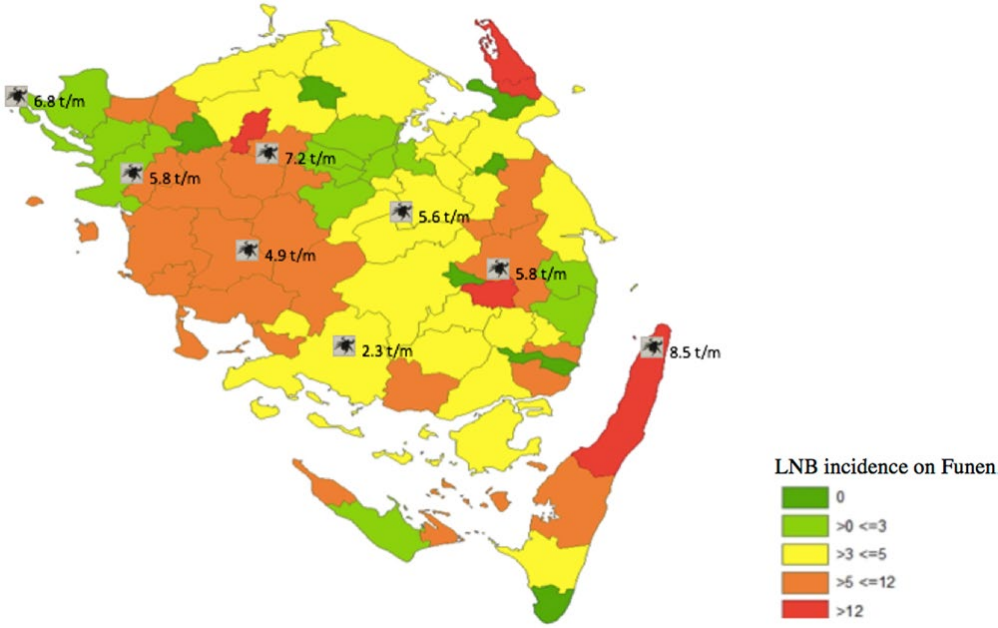
Lyme Neuroborreliosis (LNB) (per 100,000/year) incidence map of Funen, Denmark, color-coded at a zip-code level including tick density. The ticks illustrate the average tick density at individual locations collected by monthly flagging from April-November 2002 ref. ^23^ (Flagging is the technique of collecting ticks by moving a piece of fabric mounted on a stick through the vegetation for a given period of time). Software used to provide figure, ArcMap 10.6.1. The result shows a LNB “high-medium incidence-belt” (incidence up to 19.6/100,000) going south.

We found the annual LNB incidence rates on Funen varying from 2.33 (1998) to 7.93 (2006) (Fig. 3). The incidence rate at zip-code level is displayed in Fig. 4 ^22^. The ticks illustrate the average tick density at individual locations collected by monthly flagging from April-November 2002 in a previous study by Skarphedinsson^23^.

### Local spatial clustering

**Figure 5:**
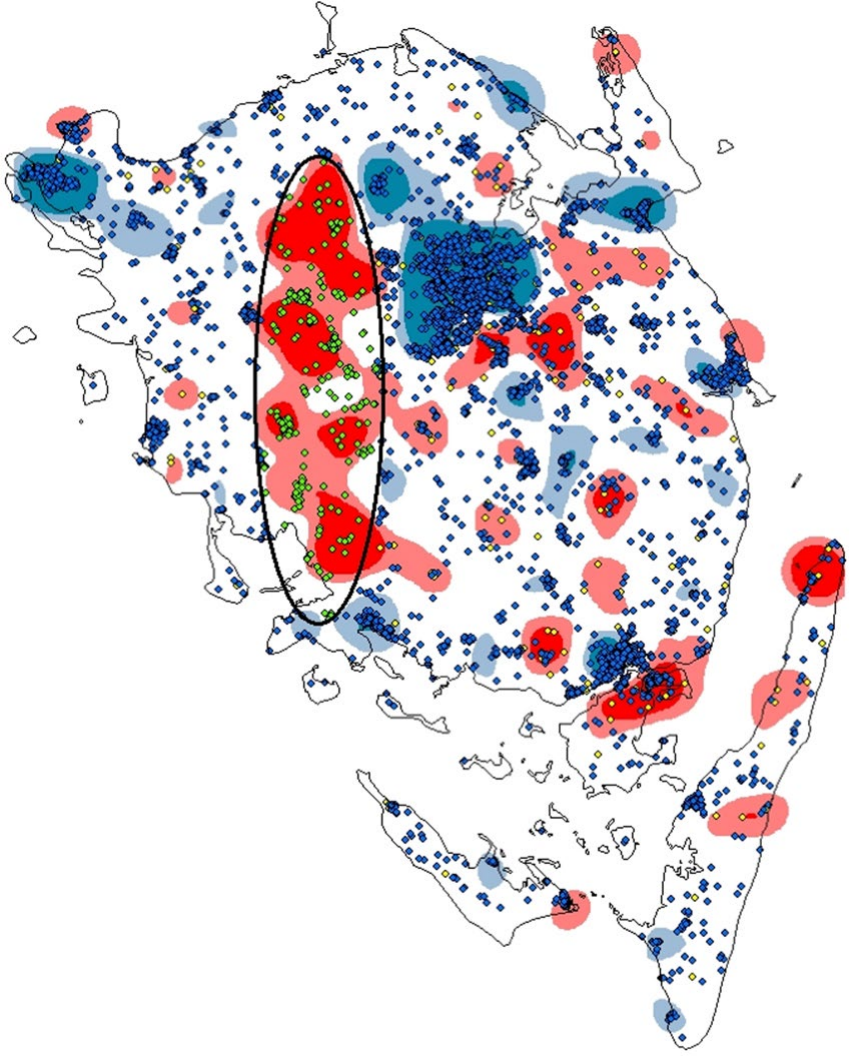
The distribution of Lyme Neuroborreliosis case-addresses (n = 401, yellow) notified on Funen, Denmark 1995–2014, and control-addresses (n = 4001, blue), with color-interpolarization technique. This is supplemented with a significant elliptic shaped cluster (hot-spot) from SaTScan analysis. The areas with domination of cases are illustrated as high density red areas, whereas the blue high density areas are dominated by controls. Green dots illustrate all containing cases and controls in the ellipse (hot-spot), and thereby a RR 2.44 for being a case (p = 0.013). Software used to provide figure, ArcMap 10.6.1.

Interpolation of the LNB-cases and controls showed areas with apparent high and low density of cases, with high incidence areas visually appearing to be correlated with the distribution of forest areas. The densely populated urban area of Odense in the middle of the main island appeared as an area with relatively few cases. West of Odense was a large elongated area with a relatively high proportion of cases (Fig. 5). The SaTScan^18^ analysis detected a significant local ellipsoid hot-spot with a relative risk of 2.44 (p = 0.013). This significant ellipse-shaped cluster overlapped the area west of Odense idicated by interpolation^24^. No other areas of apparent high or low densities of cases indicated by the IWD were detected as significant (5% significance level) clusters by the SatScan analysis.

### Distance to the nearest forest

**Figure 6:**
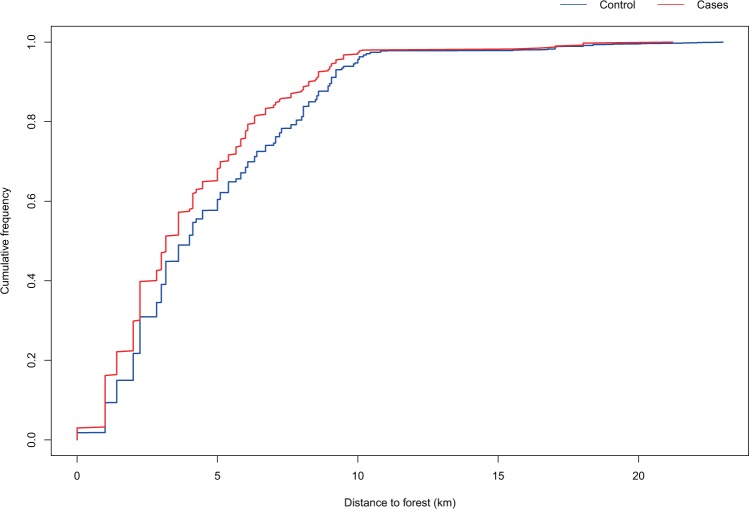
The cumulative frequency of the distance to the closest forest for Lyme Neuroborreliosis cases. Cases (n = 401) and controls (n = 4001). P-value <0.001. P-value from the logistic regression model (result section).

The cumulative frequency graph of the distance to the nearest forest illustrates a significantly shorter distance for cases compared to controls. A regression analysis found that the odds of being a case rather than a control increased with approximately 8% (Odds ratio 1.08) when moving one km closer to a forest (p < 0.001) (Fig. 6, Table 1).

**Table 1:** Results of mixed logistic regression identifying the effect of distance to forest to number of positive cases.

Parameter	Estimate	Std. error	z-value	P-value	Odds ratio	Variance	Std. Dev.
Intercept	−2.00	0.10	19.92	<0.0001	7.38		
Forest dist.	0.07	0.02	3.50	<0.001	1.08		
**Random effects:**							
Grid id						0.01	0.11

## Discussion

In this large retrospective study of a well-defined case population of LNB, we aimed to examine the temporal changes in LNB incidence. The average LNB incidence rate of 4.76/100 000 inhabitants found in our cohort was higher than previously described in a Danish setting^6,25,26^. This increase in incidence rate supports the hypothesis suggested by Dessau *et al*. that the incidence found in previous Danish studies has been underestimated^6^. Our results suggest a bias in the previous national incidence numbers due to a lack of case reporting. Since LNB can have consequences, not only for the individual patient but also socioeconomically^27^, correct reporting is essential to strengthen future LNB surveillance.

As for our aim to examine any seasonal changes in LNB incidence, we found a significant seasonal variation with most cases having their symptom-onset in July. This is earlier than previous findings in studies from our neighbouring countries where symptom-onset peak has been in August^28,29^. This finding is important for clinicians, to increase awareness of earlier seasional LNB onset.

We divided seasonal variation data into 5-year intervals to investigate if changes in the seasonal distribution could be correlated to changes in the European climate, as discussed by Lindgren *et al*. and Rizzoli *et al*.^30–32^. Seasonal distribution of casesdid not differ significantly over time during the 20-year study period. However, it is possible that 20 years of data is too short of a timeline to detect any seasonal shift in the number of monthly LNB cases due to climate change.

We aimed to identify any spatial patterns of LNB cases on Funen. We found that LNB incidence on Funen displayed a great spatial variation being higher in rural areas, particularly near forest areas. We found an elliptic shaped cluster on mid-western Funen with a significant increase in relative risk of LNB. This cluster visually appeared to be correlated to the distribution of forest areas, and may also be related to regional differences in the distribution of reservoir-hosts carrying different genospecies of *Borrelia*^33^. For the general practitioner knowledge of potiental high risk areas of LNB is of importance, and may also be utilized in prophylactic measures. These findings also underline the importance of a One Health approach to tick-borne diseases, where human disease is looked at in relation to both veterinary and the environmental factors^34^.

The overall LNB incidence in the main urban area was not significantly lower than the rural areas although the IDW interpolation indicated lower prevalence in the main city of Odense. Interestingly, a large number of cases were reported from the main Odense area. Although some of these cases may result from tick bites in urban areas it is likely that most tick-bites are aquired when visiting surrounding rural areas. If the risk of LNB is correlated to forest exposure-time (hours) alone, we would expect to observe a greater difference in relative risk between an urban and rural population. Given our results, one could ask if the risk of infection in urban areas is higher than previously assumed. This should call for an increased awareness of LNB symtoms among general practitioners and hospital docters not only in rural but also in urban areas.

The distance to the nearest forest was significantly shorter for cases compared to controls. The odds ratio of being a case increased with 7% when moving one km closer to a forest. This indicates, not unexpectedly, that the forest areas have an important impact on the risk of LNB infection.

The strengths of this study are the clear LNB case definition and complete data on all included patients. Other strengths are the robust surveillance data programs providing reliable geographical data from the study area. The process of adjusting the control addresses might have given a small selection bias. The authors do not know if every address included in the control group was inhabited. However, we do know that the total percentage of uninhabited addresses on Funen was low at time (6,4%)^35^, which limits this potential bias.

Future studies in this area should focus on identifying better local data-classification of landcover variables and variables describing temporal and spatial variation in animal abundance. This will help predicting areas with a high possibility of LNB case presence, as well as regional incidence trend variations as previously discussed by Messier *et al*.^36^. These variables can be used in models predicting areas with a higher risk of LNB infection and increase the accuracy of risk maps. A future local risk-map could be used as a tool by general practitioners in high-risk areas as well as increase awareness of LNB and LNB symptoms in people living in these areas. This could potentially shorten diagnostic and treatment delay and thereby reduce the risk of longterm sequelae^7,37^. Collaborative preventive actions on a European level creating a European model for predicting LNB high-risk areas would be of public health interest. Planned future changes in agriculture affecting land and forest areas could then be analysed in regards to its impact on known environmental variables e.g. roe deer density that increase/decrease the risk of tickborne infections, especially in locations close to inhabited areas.

In conclusion, we found a clear seasonal pattern of distribution of LNB cases, but no seasonal changes over the 20-year study period. we identied a significant clustering of LNB-cases in the mid-western part of the island of Funen, which represents an area with increased risk of LNB. The LNB cases resided significantly closer to forests compared with controls, indicating greater exposure to *I. ricinus* in forest areas.

## Supplementary information

Supplementary information.
