# Supplementary material for: Spatial and temporal patterns of Lyme Neuroborreliosis on Funen, Denmark from 1995–2014
Source: Sci Rep. 2020 May 8;10:7796. doi: 10.1038/s41598-020-64638-5 (PMC7210281; doi:10.1038/s41598-020-64638-5)

Spatial and temporal patterns of Lyme Neuroborreliosis on Funen, Denmark from 1995 – 2014.

Authors: Amalie Muus Andreasen[1*], Petter Bart Dehlendorff[1*], Fredrikke Christie Knudtzen[1,2], Rene Bødker[3], Lene Jung Kjær[3], and Sigurdur Skarphedinsson[1,2]

Affiliations

1: Department of Infectious Diseases, CCEVI – Clinical Centre of Emerging and Vector-borne Infections, Odense University Hospital, Winsloews vej 4, 5000 Odense C, Denmark

* These authors contributed equally to this manuscript.

2: Clinical Institute, University of Southern Denmark, Campusvej 55, 5230 Odense, Denmark

3: Department of Veterinary and Animals Sciences, University of Copenhagen, Frederiksberg, Denmark

Supplementary material

Supplement S1

The geographical distribution of forests [a] on Funen, Denmark, illustrated with CORINE landcover data (from 2006).

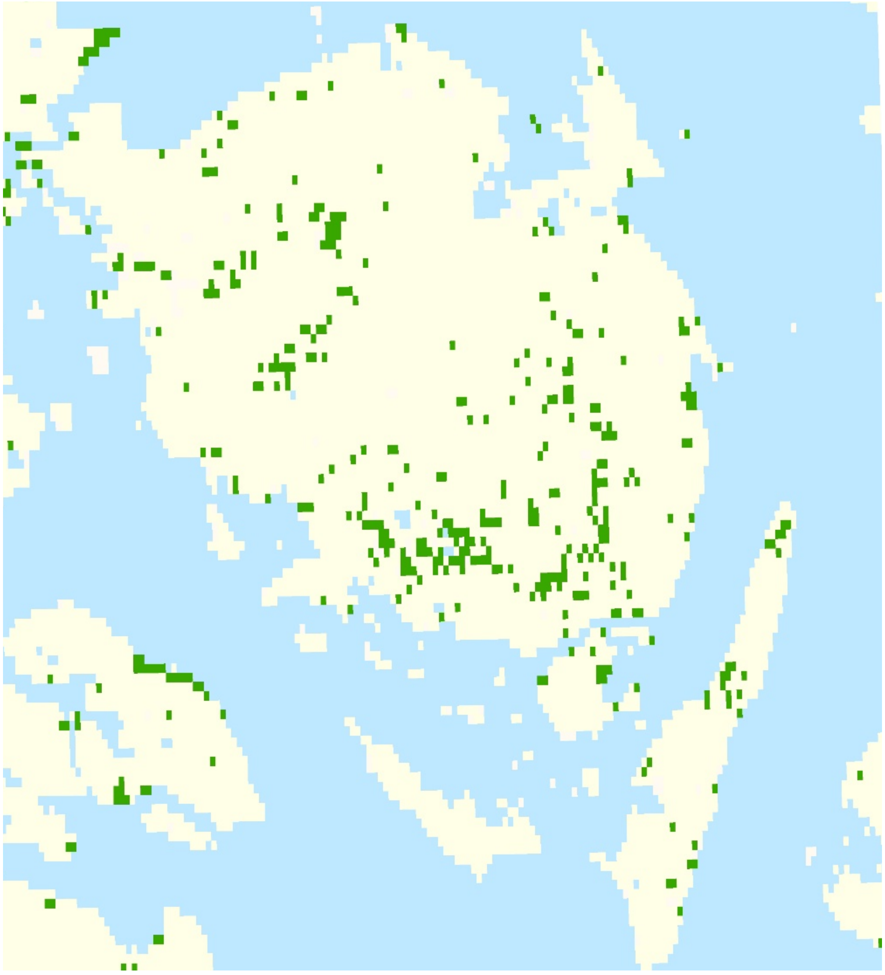

[a] Forest defined as: Landcover codes; broad leaved forest (code 23), coniferous forest (code 24) and mixed forest (code 25). All forest codes are illustrated as the same type of green pixels. Software used to provide figure, ArcMap 10.6.1

Supplement S2

Grid overlay of Funen with cases (red) and controls (blue) used in this study. The ID of each grid cell was used as a random effect in a mixed model logistic regression to account for spatial autocorrelation.

Software used to provide figure, ArcMap 10.6.1

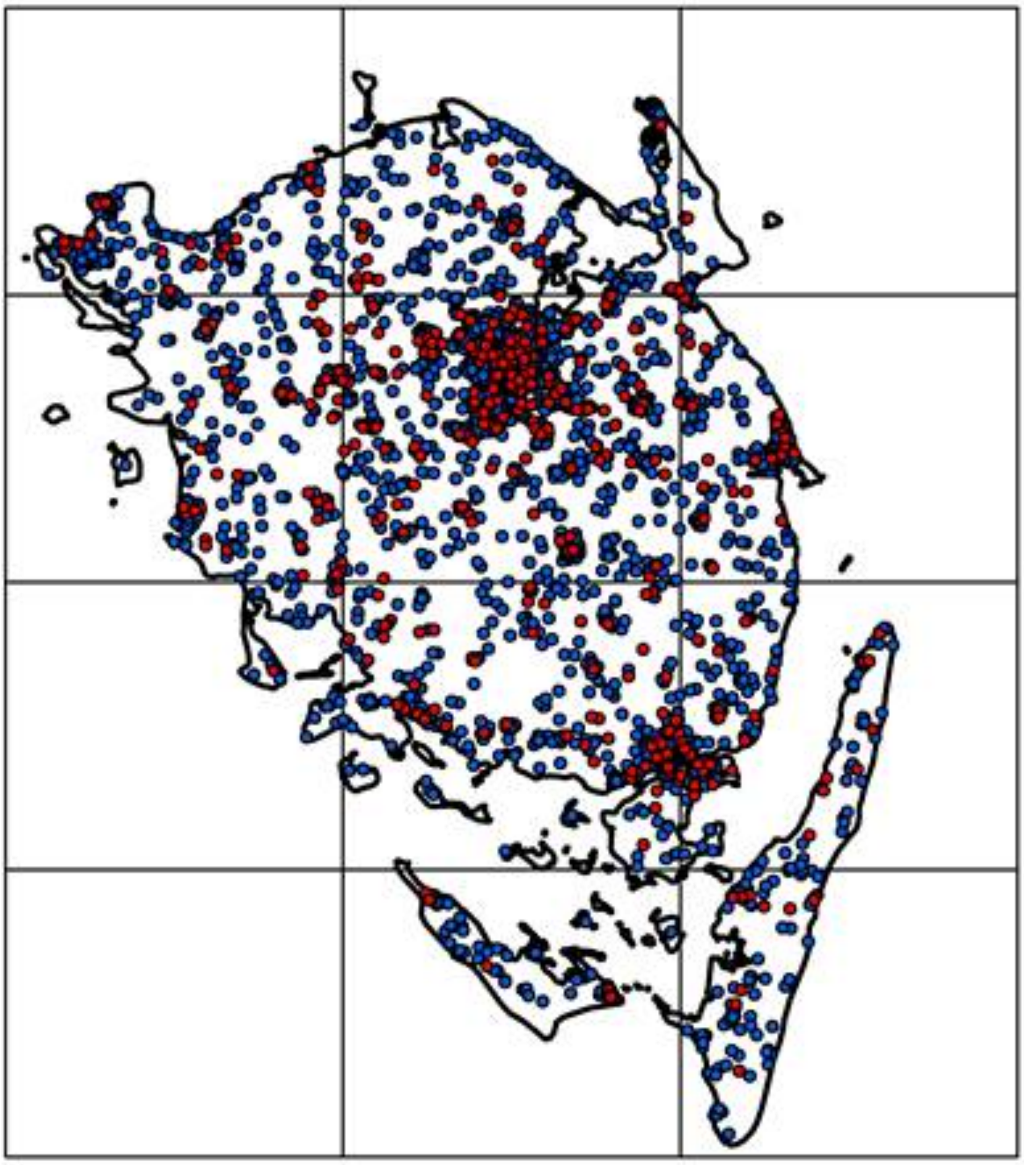

Supplement: Supplementary file 1 — Supplementary information. [file 41598_2020_64638_MOESM1_ESM.pdf]
